# Surface damage of bovine articular cartilage-off-bone: the effect of variations in underlying substrate and frequency

**DOI:** 10.1186/s12891-018-2305-2

**Published:** 2018-10-24

**Authors:** Humaira Mahmood, Duncan E. T. Shepherd, Daniel M. Espino

**Affiliations:** Department of Mechanical Engineering, University of Birmingham, B15 2TT, Birmingham, UK

**Keywords:** Articular cartilage, Bone mineral density, Damage, Frequency, Mechanical loading, Osteoarthritis

## Abstract

**Background:**

Changes in bone mineral density have been implicated with the onset of osteoarthritis, but its role in inducing failure of articular cartilage mechanically is unclear. This study aimed to determine the effect of substrate density, as the underlying bone, on the surface damage of cartilage-off-bone, at frequencies associated with gait, and above.

**Methods:**

Bovine articular cartilage samples were tested off-bone to assess induced damage with an indenter under a compressive sinusoidal load range of 5–50 N at frequencies of 1, 10 and 50 Hz, corresponding to normal and above normal gait respectively, for up to 10,000 cycles. Cartilage samples were tested on four underlying substrates with densities of 0.1556, 0.3222, 0.5667 and 0.6000 g/cm^3^. India ink was applied to identify damage as cracks, measured across their length using ImageJ software. Linear regression was performed to identify if statistical significance existed between substrate density, and surface damage of articular cartilage-off-bone, at all three frequencies investigated (*p* < 0.05).

**Results:**

Surface damage significantly increased (*p* < 0.05) with substrate density at 10 Hz of applied frequency. Crack length at this frequency reached the maximum of 10.95 ± 9.12 mm (mean ± standard deviation), across all four substrates tested. Frequencies applied at 1 and 50 Hz failed to show a significant increase (*p* > 0.05) in surface damage with an increase in substrate density, at which the maximum mean crack length were 3.01 ± 3.41 mm and 5.65 ± 6.54 mm, respectively. Crack formation at all frequencies tended to form at the periphery of the cartilage specimen, with multiple straight-line cracking observed at 10 Hz, in comparison to single straight-line configurations produced at 1 and 50 Hz.

**Conclusions:**

The effect of substrate density on the surface damage of articular cartilage-off-bone is multi-factorial, with an above-normal gait frequency. At 1 Hz cartilage damage is not associated with substrate density, however at 10 Hz, it is. This study has implications on the effects of the factors that contribute to the onset of osteoarthritis.

## Background

The principal function of articular cartilage is to allow for ease in the kinetics of two connecting ends of the bones in contact [1, 2]. Cartilage prevents high stress concentrations which would be expected to occur through bone to bone contact and provides low friction articulation, aided by a surface roughness of 80–170 nm [3]. Osteoarthritis (OA) involves articular cartilage deficit and is progressive, such that joint motion becomes more painful with time [4]. Globally, OA is reported as the highest occurring joint health condition [5]. Alterations in the underlying bone are key to diagnosing OA, illustrated by the concept of an enhanced subchondral bone stiffness being associated with advances in cartilage impairment [6]. However, the link between destruction of cartilage and changes in subchondral bone are less clear, focusing on associating the relationship between a high bone mineral density (BMD) and cartilage degradation [7, 8]. This relationship is further associated by the development of radiographic knee OA [9], and with increased cartilage volume [10] and cartilage thickness [11] during the early stages of OA.

The mechanical behaviour of articular cartilage appears to differ when off-bone and on-bone. For example, off-bone articular cartilage is more capable of dissipating energy than on-bone articular cartilage [12, 13], with the restrictive behaviour of the underlying bone constraining articular cartilage [12–14]. However, there may be a direct effect by the underlying bone on the mechanical characteristics of the overlying articular cartilage [15]. This has been recently shown by the correlation between BMD and the loss modulus of articular cartilage [16]. Further, the correlation between the effective cartilage tangent modulus and the Young’s modulus of its underlying substrate, such that damage to cartilage via impact loading has occurred at a decline in effective cartilage and substrate modulus, representing cartilage damage at a lowered BMD [17].

Damage experienced by articular cartilage has been linked to the mechanism of loading, such as the effects of loading rates [13, 18], impact loading [17, 19], and frequency independent of load [2, 20, 21]. Further, the effects of hydration [22] as well as rapid heel-strike [2, 13, 20, 21] have also been associated with cartilage damage. However, it is unknown whether there is a direct mechanical link of a variation in frequency, between the damage experienced by articular cartilage and the density of its underlying subchondral bone.

Therefore, the aim of this current investigation was to assess, experimentally, whether substrate density affects the surface damage of bovine articular cartilage-off-bone, with a variation in applied frequency associated with normal gait; 1 Hz, and above normal gait; 10 and 50 Hz [2, 23, 24]. Surface damage was evaluated as total crack length, identified with the application of India ink, following on from comparison to the cartilage-off-bone specimen photographed prior to testing, for clear damage detection.

## Methods

### Preparation of specimens

**Fig. 1:**
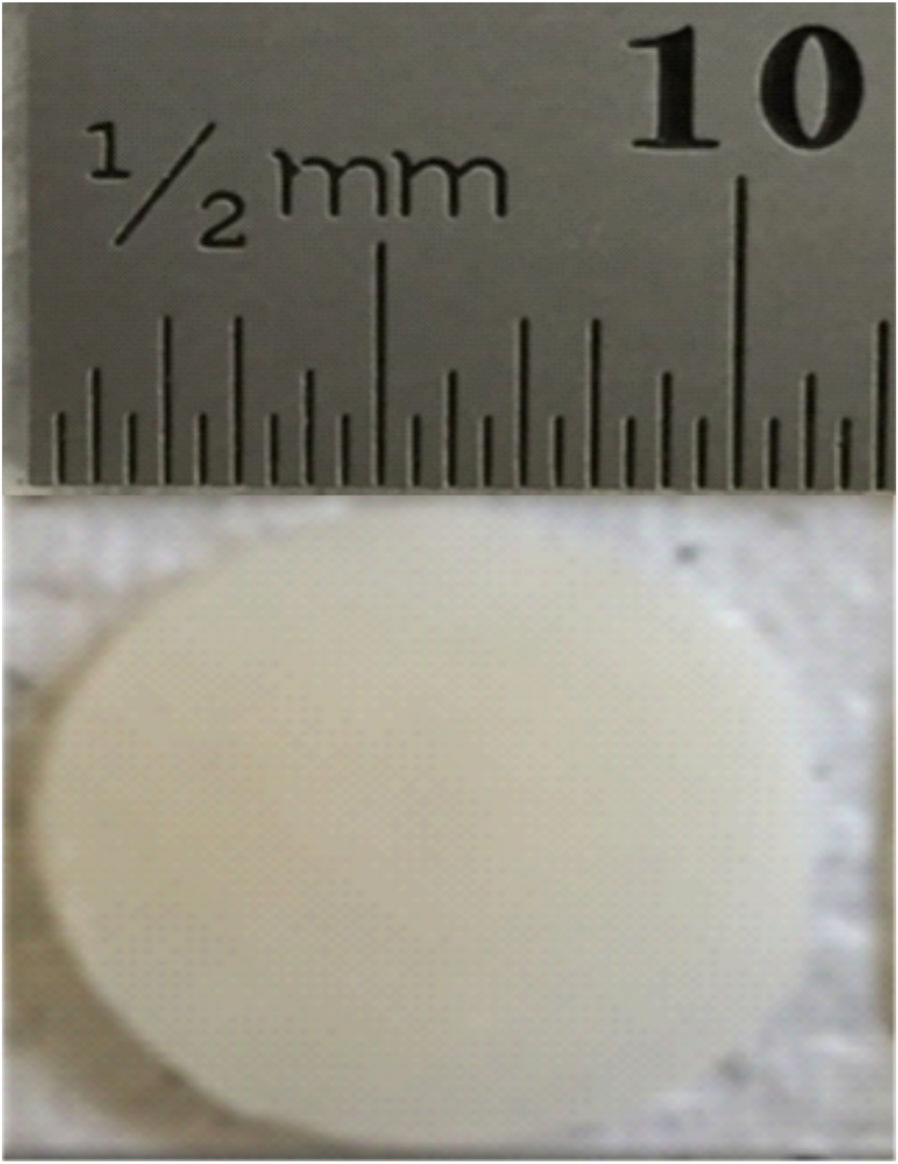
Representative image captured of bovine articular cartilage specimen, prior to testing on confirmation of the absence of damage with India ink. Scale bar is included (mm)

Twelve bovine humeral heads were obtained from a supplier (Dissect Supplies, Kings Heath, Birmingham, UK) from animals of maximum 30 months old at slaughter. Bovine articular cartilage was selected based on the previously established relationship of the frequency-dependent viscoelastic properties, consistent with that of human articular cartilage [25]. The humeral heads were covered in tissue paper, coated with Ringer’s solution prepared to a full strength mass concentration by dissolving 4.83 g of Ringer’s tablets (Oxoid Ltd., Hampshire, UK), per 500 ml of distilled water, and separately stored in double heat-sealed plastic at − 40 °C [13, 23]. Bovine humeral heads were thawed at room temperature for testing preparation [25]. The freeze-thaw process does not affect the mechanical properties of articular cartilage [26]. India ink (Daler-Rowney, Bracknell, UK) was used to ensure that humeral head cartilage surfaces were free from lesions ahead of testing [13, 21, 27]. For this procedure, the entire humeral head surface was covered with India ink so that on removal (rinsing) any defects could be identified; a commonly employed technique [13, 21, 27]. This allowed the coring procedure to take place at intact regions only. Further, on harvesting of each cartilage core, India ink was applied to each specimen, rinsed off, and an image captured for comparing damage post-testing. A representative image of a specimen captured before testing (but following application/removal of India ink) is displayed in Fig. 1.

### Cartilage-off-bone specimens

To prepare off-bone cartilage cores, a hand cork-borer with an *on face* diameter of 10.5 mm was used to create circular dents on the surface of the articular cartilage, and through to the underlying bone. Following on from confirmation with the use of India ink of undamaged regions for coring, caution was taken during the harvesting process to attain undamaged cartilage specimens. Further, the absence of damage was confirmed via observation following the use of India ink post-harvesting. The locations selected were identical for all specimens, namely at the central region, ensuring the cartilage surface was complete and undamaged [25]. A surgical scalpel with blade size 10A (Swann-Morton, Sheffield, UK) was used to isolate the cartilage core from the underlying subchondral bone [12, 13, 17, 25, 28]. On harvesting of the cartilage cores from the underlying bone, they were immediately immersed in Ringer’s solution to prevent dehydration. Furthermore, the specimens were extracted on the day of testing; curling was not observed on any tissue harvested. To measure thickness, the cartilage core was held with a Vernier calliper at its centre; care was taken to avoid compression of the cartilage specimen. The thickness of the cartilage specimens measured prior to testing was 0.99 ± 0.004 mm (mean ± standard deviation), and the diameter was 10.9 ± 0.210 mm, as measured using a Vernier calliper (Draper Tools Ltd., Hampshire, UK). Six off-bone bovine articular cartilage cores were removed from each of twelve humeral heads, resulting in 72 individual specimens of off-bone cartilage. The specimens were immersed in Ringer’s solution for 30 min [29] prior to testing [25].

### Substrate design and mechanical loading

Specimens were positioned on the substrate blocks (Sawbones, Washington, USA) for testing. Four Sawbone densities were tested, which were 0.1556, 0.3222, 0.5667 and 0.6000 g/cm^3^. The substrate with the lowest density, substrate one, was used as an osteoporotic representation of bone [30, 31] whereas the highest density, substrate four, was closer to that of cancellous bone [32]. The Sawbone was prepared with a vertical bandsaw (Startrite Volant 24, UK) into blocks with dimensions 30 mm × 30 mm × 10 mm. The Young’s modulus of each substrate was obtained from compression tests using a Bose Electroforce 3300 testing machine run using Bose WinTest software (Bose Corporation, ElectroForce Systems Group, Minnesota, USA; updated to: TA Instruments, New Castle, DE, USA), derived from the slope of the stress-strain curve calculated from the force-displacement plot, at 0.02 mm/s with compression from a metal plate, 81 mm in diameter onto the substrate block (Table 1).

**Table 1:** Material properties of the four underlying substrates utilised to represent varied bone mineral densities positioned beneath each cartilage-off-bone core during testing, allowing for investigation of the effect of substrate density on the damage to articular cartilage

Substrate	Density (g/cm^3^)	Mass (g)	Young’s Modulus (*E*) (MPa)	Volume (cm^3^)	Poisson’s ratio
1	0.1556	1.4	435	9	0.30
2	0.3222	2.9	810	9	0.30
3	0.5667	5.1	1238	9	0.30
4	0.6000	5.4	2861	9	0.30

**Fig. 2:**
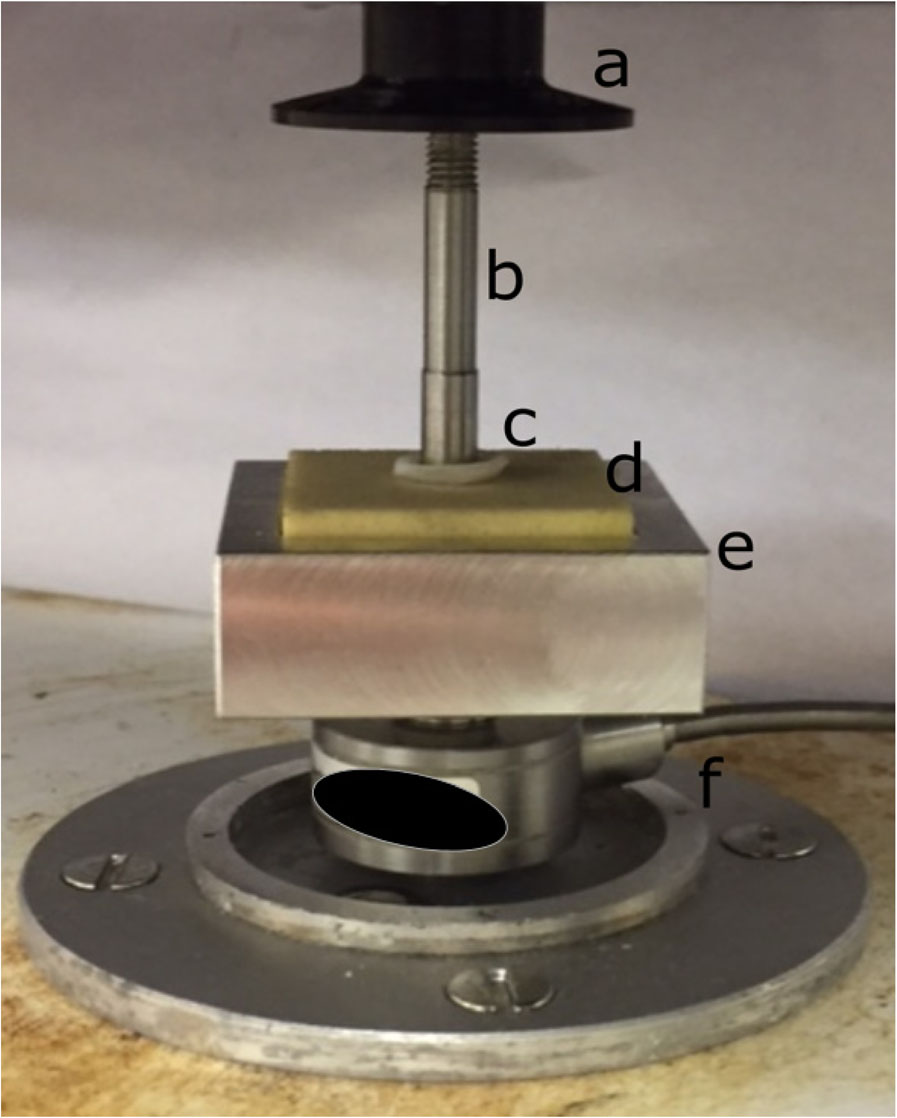
Example set-up at actuator (**a**) for mechanical testing with stainless-steel indenter (**b**) at off-bone cartilage specimen (**c**) and lowest density underlying substrate (**d**), within customised test rig (**e**). Cartilage off-bone specimen is positioned above the substrate. Stainless-steel indenter is lowered onto the cartilage-off-bone specimen for testing, movement operated with testing machine. Load cell component (**f**) for experimental load control

A square aluminium test rig was designed with dimensions 41 mm × 41 mm × 16 mm into which the Sawbone substrate was placed. A stainless-steel flat circular faced indenter, with a 0.5 mm bevelled edge to avoid stress concentration at its edges, 5.2 mm in diameter [2] was fixed to the actuator of the testing machine and used to induce damage through indentation to the surface of the cartilage-off-bone specimen. This procedure was similar to a previous study for on-bone cartilage [2]. A Bose ElectroForce 3200 testing machine controlled via the Bose WinTest 4.1 software (Bose Corporation, ElectroForce Systems Group, Minnesota, USA; updated to: TA Instruments, New Castle, DE, USA) was used for testing. The stainless-steel indenter was descended onto the cartilage specimen at the point of testing (Fig. 2).

Six cartilage specimens were tested per substrate, at a given frequency. The cartilage specimens were manually positioned on the underlying substrate, without the use of adhesive treatment prior to testing. Specimens were not observed to move during testing. This placement replicates the effect of the soft-on-hard construct but may not replicate the restriction of collagen at the cartilage calcified zone, due to the removal of the underlying bone [12]. A sinusoidally varying force under unconfined compression between 5 and 50 N was applied to the specimens for 10,000 cycles [2]. Repeated loading has previously been found to induce disruption to the articular cartilage surface [1, 2, 33]. Frequencies of 1, 10 and 50 Hz were applied to the specimens, for a completion of 72 individual tests. At 5000 cycles, the halfway point during testing, the articular cartilage-off-bone specimens were irrigated with Ringer’s solution [20, 21], to ensure hydration of the cartilage. Further, recent work has also confirmed the absence of a significant difference on the dynamic viscoelastic behaviour of articular cartilage, when tested for short periods in either air or Ringer’s solution [13].

### Quantification of changes to the cartilage surface

Pictures were taken of each specimen before and after testing for clear observation of the damage induced after testing. India ink was used to identify alterations to the cartilage-off-bone specimen following testing, in addition to the sample prior to testing for damage absence confirmation [2, 27, 33]. For evaluation of the damage present, ImageJ software (version 1.48, Rasband, W.S., U.S. National Institutes of Health, Bethesda, Maryland, USA) was used with calibration of the image via a scale bar. The area of indentation (mm^2^) and crack length (mm) were analysed as two separate conditions for each specimen. Image measurements were repeated twice per sample and the mean value reported. Firstly, the area indented following testing with the indenter was highlighted (i.e. a non-damaged measurement), with use of the free-hand tool on the ImageJ software. Secondly, the length of cracks was measured using an existing method, and this was considered as a damage measurement [2].

### Data analysis

Sigmaplot Version 12.0 (Systat Software Inc., London, UK) was used to perform regression analysis on the relationship between the four substrates of varying densities and the following parameters: the area of indentation; crack length; damage, at all frequencies tested; 1, 10 and 50 Hz. A linear regression fit was assessed as the most appropriate representation of the empirical relationships derived (*p* < 0.05).

## Results

### Surface assessment

**Fig. 3:**
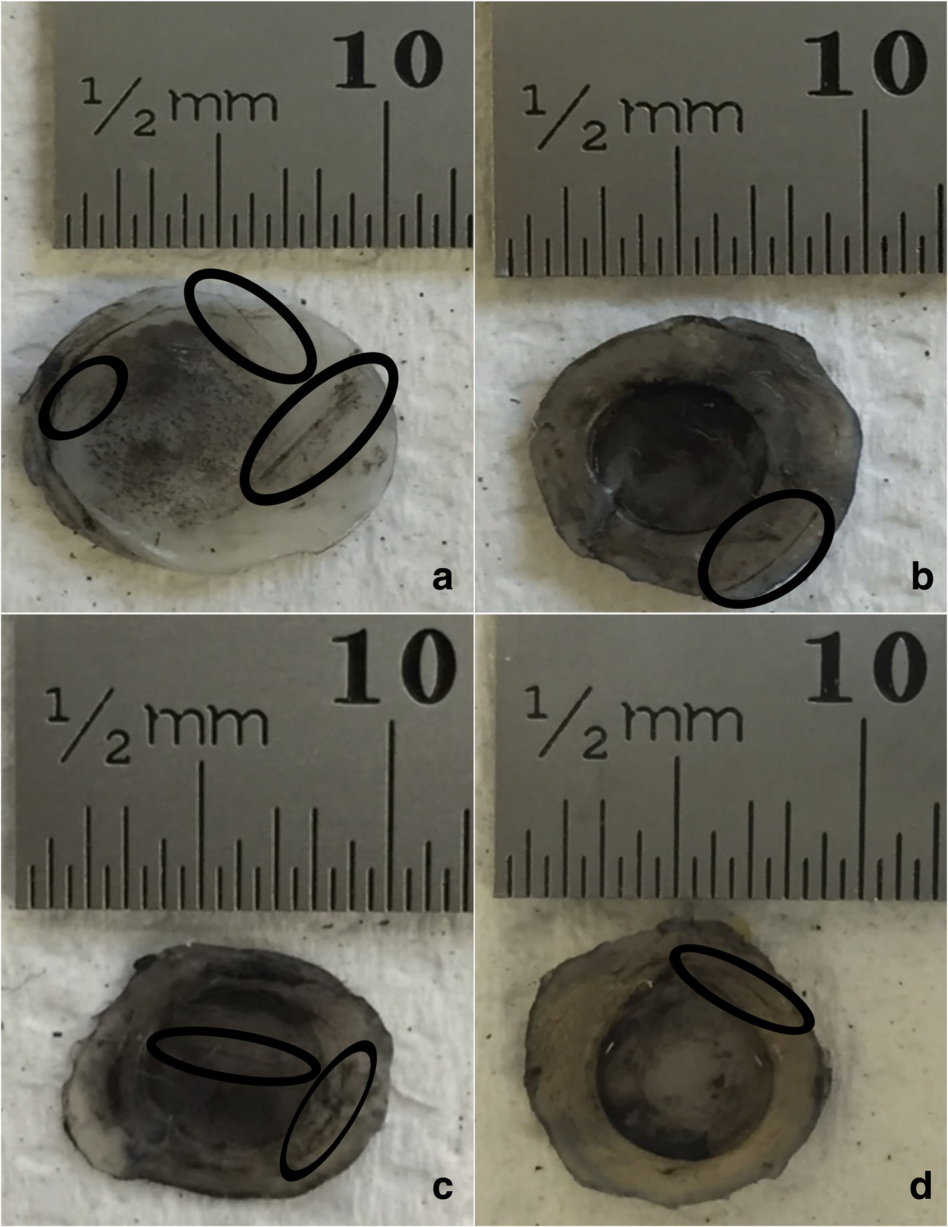
Representative images of bovine articular cartilage-off-bone samples after testing, at 1 Hz frequency of loading. Image **a**-**d** display a sample result at substrates 1–4, respectively. Damage as cracks and indentation were identified with application of India ink. Cracks formed are highlighted with the black ellipse for clear observation. Indentation can be observed across most of the cartilage-off-bone specimen surface, at this frequency of loading. Scale bar (mm) included for quantifying results

**Fig. 4:**
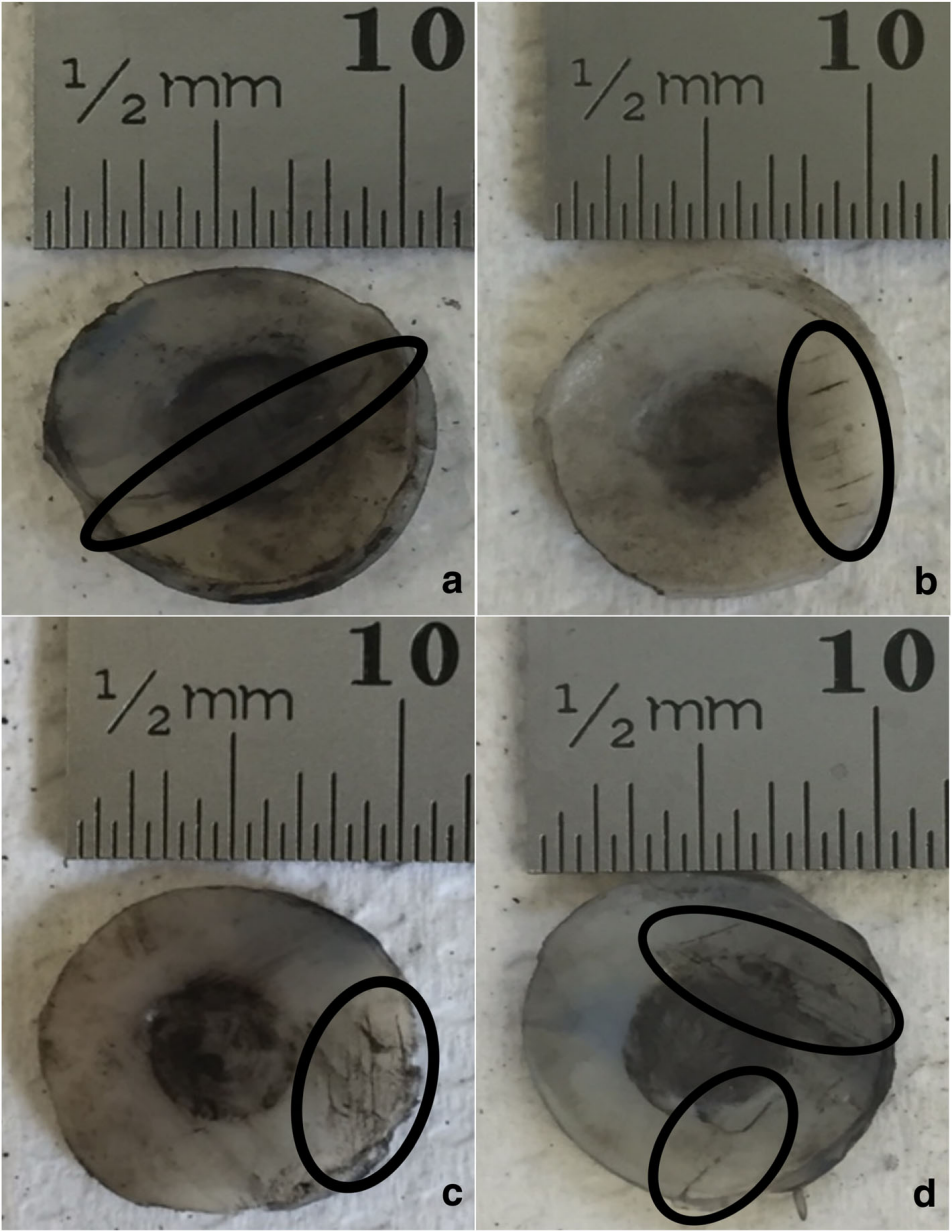
Representative images of bovine articular cartilage-off-bone samples after testing, at 10 Hz frequency of loading. Image **a**-**d** display a sample result at substrates 1–4, respectively. Damage as cracks and indentation were identified with application of India ink. Cracks formed are highlighted with the black ellipse for clear observation, notably of multiple parallel straight-lines. Scale bar (mm) included for quantifying results

**Fig. 5:**
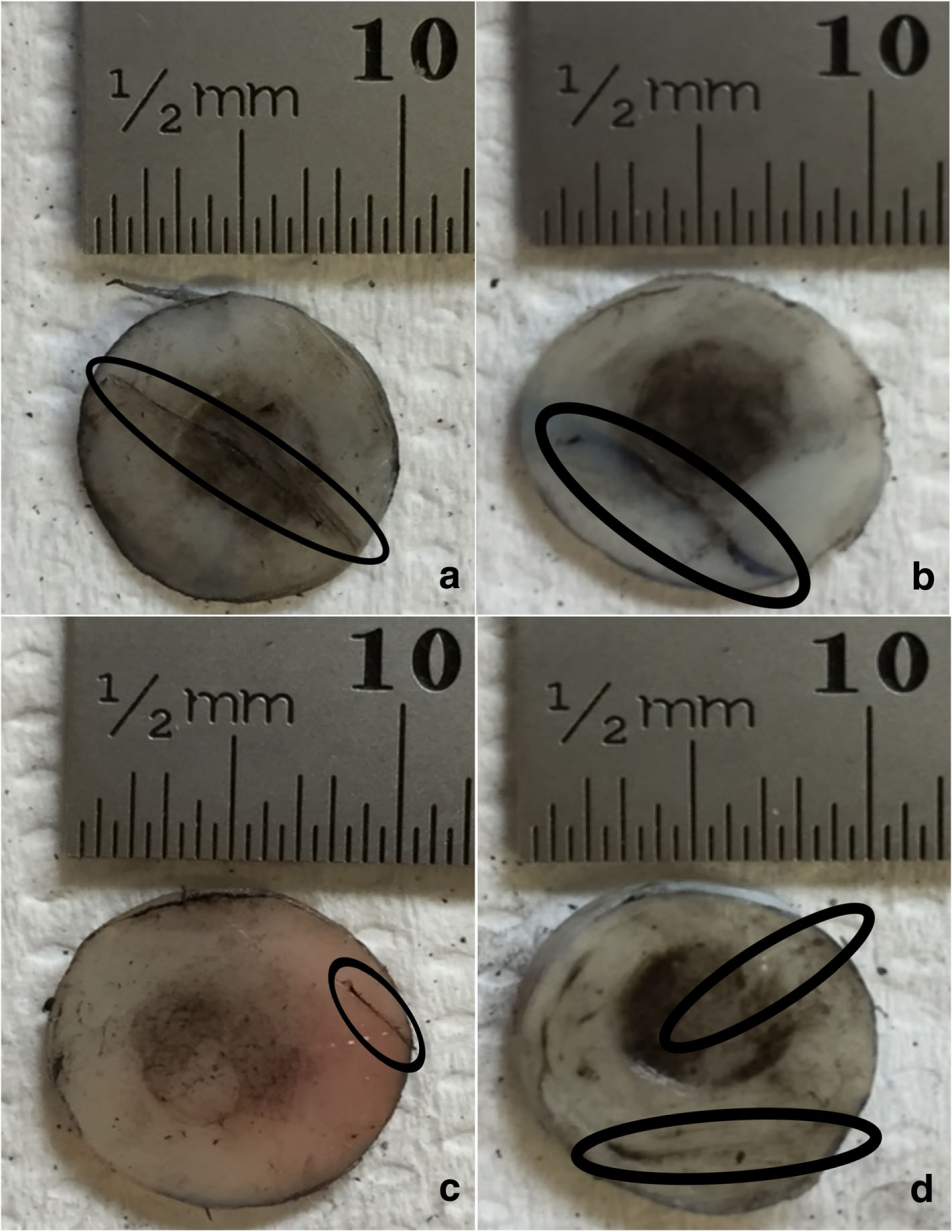
Representative images of bovine articular cartilage-off-bone samples after testing, at 50 Hz frequency of loading. Image **a**-**d** display a sample result at substrates 1–4, respectively. Damage as cracks and indentation were identified with application of India ink. Cracks formed are highlighted with the black ellipse for clear observation, notably of single-line configurations of varying lengths. Scale bar (mm) included for quantifying results

**Fig. 6:**
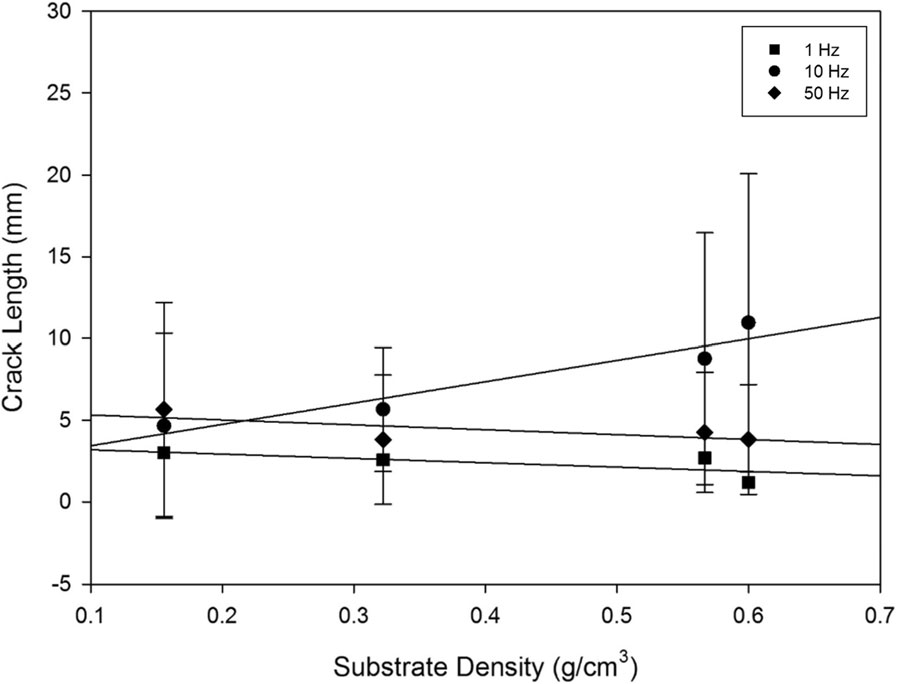
Mean crack length plotted with substrate density at 1, 10 and 50 Hz for off-bone articular cartilage, represented by the square, circle and diamond, respectively. Linear regression displayed by eq. (1) fit the data at R^2^ values of 0.485, 0.909 and 0.524 at 1, 10 and 50 Hz, respectively. Error bars represent standard deviations

**Fig. 7:**
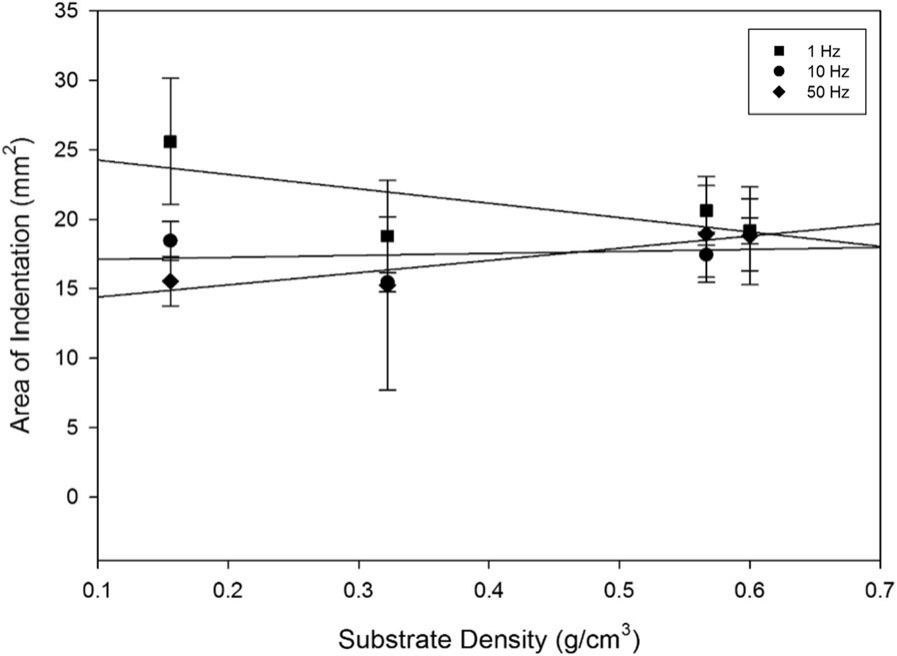
Mean area of indentation plotted with substrate density at 1, 10 and 50 Hz for off-bone articular cartilage, represented by the square, circle and diamond, respectively. Linear regression displayed by eq. (1) fit the data at R^2^ values of 0.487, 0.0386 and 0.851 at 1, 10 and 50 Hz, respectively. Error bars represent standard deviations

Representative images of cartilage specimens after testing are shown for 1, 10 and 50 Hz, in Fig. 3, Fig. 4 and Fig. 5, respectively, at all four substrates investigated. The damage observed as crack formation is clearly indicated with a black outlined ellipse. Figure. 6 and Fig. 7 display the relationship between substrate density and the mean crack length and mean indented area, respectively, at all three frequencies investigated.

Crack length was significantly correlated to substrate density at 10 Hz (*p* < 0.05) (Fig. 6, Table 2), however, it was not significantly associated with substrate density at 1 or 50 Hz (*p* > 0.05) (Fig. 6, Table 2). The combined variables of frequency at 10 Hz with substrate density led to an increase in crack length with density (*p* < 0.05) (Fig. 6, Table 2). This relationship between the effects of frequency and substrate density, on the crack length of off-bone articular cartilage is best described by a linear curve as represented by eq. (1):

1 $$ c\kern0.3em =\kern0.3em D\kern0.3em +\kern0.3em AB $$

**Table 2:** Statistical details derived from mean crack length and substrate density plots of Fig. 6, at frequencies 1, 10 and 50 Hz. A and D are the constants from the curve fits. R^2^, the squared correlation coefficient indicates the extent of the data and regression line fit. A *P*-value less than 0.05 confirms statistically significant data, as displayed at 10 Hz of loading frequency

Frequency (Hz)	Total crack length linear fit details			
	A	D	R^2^	*P*
1	(−)2.652	3.454	0.485	0.304
10	(+)13.077	2.124	0.909	0.047
50	(−)2.999	5.612	0.524	0.276

where *c* is the crack length mean total, *A* is the gradient of the slope, *B* is substrate density and *D* is the intercept; *D* and *A* are empirically derived constants. The associated details are summarised in Table 2, at all three frequencies tested.

Frequency of loading in combination with substrate density had no effect on the area of indentation when samples were tested at 1, 10 and 50 Hz (*p* > 0.05) (Fig. 7). The effect of frequency alone, on the area of indentation, however, did demonstrate a negative correlation between an increase in frequency from 1 to 50 Hz, and the indented area (Fig. 7). The post-test recovery following the testing of the cartilage specimens varied for each frequency. Testing at 1 Hz of frequency resulted in the least recovery of the articular cartilage specimen; with a larger indented area observed. This is represented by the observations where the indented area, as clearly highlighted by the black circular staining of India ink, extended to the majority of the cartilage specimen surface at 1 Hz (Fig. 3), in comparison to the lesser surface indented at 10 (Fig. 4) and 50 Hz (Fig. 5).

Sample images displayed at 1 Hz (Fig. 3) illustrate cracks that were of a single-line configuration, notably through the specimen periphery, parallel to the specimen circumference, observed at a maximum mean of 3.01 mm (Fig. 6; Table 3). As the applied frequency at testing increased to 10 Hz, mean crack length at this frequency increased to its maximum by 7.94 mm (Fig. 6; Table 3).

**Table 3:** Mean total crack length values with standard deviation for cartilage-off-bone samples, following testing, at all frequencies and substrates investigated, as a combined study

Frequency (Hz)	Mean total crack length (mm) ± SD			
	Substrate 1	Substrate 2	Substrate 3	Substrate 4
1	3.01 ± 3.41	2.57 ± 2.42	2.69 ± 2.66	1.19 ± 1.63
10	4.65 ± 5.66	5.65 ± 3.80	8.76 ± 7.70	10.95 ± 9.12
50	5.65 ± 6.54	3.80 ± 3.95	4.25 ± 3.68	3.82 ± 3.36

Representative images of 10 Hz in applied frequency at each substrate (Fig. 4), illustrate crack formation predominantly observed as multiple parallel straight-line arrangements, of various lengths, similar to previous studies [1, 2], commonly observed at the periphery of the cartilage specimen. At 50 Hz, the maximum mean crack length reduced by 5.30 mm from 10 Hz (Fig. 6; Table 3), where crack formation was primarily located across the diameter of the sample of single-line conformations, of various lengths, parallel to the specimen circumference (Fig. 5).

## Discussion

In this study the measured surface damage experienced by articular cartilage was independent of substrate density, as a single variable. Increased damage due to greater energy absorption is expected with a stiffer substrate, however, this was not observed statistically, for substrate density alone, using our experimental protocol (with articular cartilage off-bone). Instead, it was demonstrated that a combined effect of substrate density, and a specific loading frequency of 10 Hz, led to an increase in surface damage (total crack length) in the cartilage. Thus, it is suggested that the combination of BMD and above normal gait frequencies (e.g. 10 Hz), predisposes articular cartilage to damage.

It is worth highlighting the importance of the particular application of 10 Hz in frequency. Within the region of, and beyond, 10 Hz, it is thought that cartilage enters a glass transition phase [21]. Therefore, a change in cartilage material properties is noticed from behaving as a deformable (‘soft’) material to one which is hard but brittle [24]. Due to this alteration in the physical behaviour of articular cartilage, it may affect the extent of damage. This is supported by the results shown in this study, as maximum cartilage damage is observed at 10 Hz, in comparison to 1 and 50 Hz. The reduction in damage observed above the application of 10 Hz, such as 50 Hz as in this study, could be due to the recently established relationship such that the loss stiffness of off-bone articular cartilage is dependent upon frequency [13]. Thus, articular cartilage may be better able to disspiate energy at 50 Hz which might explain why, off-bone, the extent of damage to the tissue is reduced as compared to 10 Hz of loading.

The multi-factorial finding from this study that an increase in substrate density combined with 10 Hz increases surface damage, corresponds to the advancement in damage of cartilage during OA which may progressively worsen during a remodelling process [6]. Further, damage of cartilage during OA may relate to the remodelling process directly, rather than purely due to alterations in the stress distribution between cartilage and its underlying subchondral bone following a change in bone density. Bone remodelling is defined by the hypothesised relationship between impulse loading of the bone at the joint experiencing fracture to result in a stiffened base for the cartilage, therefore, exposing the cartilage to greater stress and enhancing its rate of damage development [6]. The process of remodelling weakens cartilage [34], so that per load it would degrade with the influence of an above normal gait frequency, independent of the effect of a high BMD alone to induce cartilage damage at 10 Hz. Therefore, in the case where an individual experiences the combined effect of an above gait-heel strike with a high BMD, this collective relationship per load may enforce the process of bone remodelling to mechanically become self-propagating as regards damage. It is also worth noting there are additional factors that relate to the development and progression of OA, including obesity [35, 36], the suggested effect of leptins on chondrocyte behaviour [37] and the role of leptins in Matrix metalloproteinases degrading collagen within the extracellular matrix [38, 39].

The peak stress induced in this study was 2.88 MPa, greater than the stress at approximately 1–1.7 MPa within the knee and hip while walking [40], thus, encouraging damage. Damage was induced on the surface of the articular cartilage at 1, 10 and 50 Hz, corresponding to frequencies associated with gait and above, consistent with previous studies [2, 20, 21]. With particular attention to the effects of frequency, excluding substrate density, the data shows mean crack length of off-bone articular cartilage increased from 1 to 10 Hz. These findings are consistent with a previous study for on-bone articular cartilage, with an increase in frequency [2, 20, 21]. However, in our current study for off-bone cartilage there was no increase in damage when loading at 50 Hz, unlike for the on-bone study of loading [2, 20, 21]. Thus, it is worth noting the differences in the behaviour of cartilage on- and off-bone, as a result of the presence or absence, respectively, of the restraining effect provided by the underlying bone [12]. This may be due to the loss stiffness being frequency-dependent for off-bone articular cartilage [13], but not for on-bone cartilage [24]. Therefore, off-bone articular cartilage is more able to dissipate energy potentially preventing damage to the cartilage itself (i.e. via dissipating the energy through the formation of cracks); this ability to dissipate energy is greater at higher frequencies, particularly at 50 Hz, potentially reducing the extent to which cartilage undergoes damage at 50 Hz (which may not happen when cartilage is on-bone).

At 1 Hz, previous work demonstrates mean total crack length close to 1 mm at the highest tested load of 160 N for on-bone cartilage [2]. At 1 Hz in this study, for off-bone cartilage, with the peak tested load at 50 N, results show a maximum mean crack length at 3.01 mm. At 10 Hz, previous work determines a mean crack length close to 2.4 mm for on-bone cartilage at the maximum load [2], whilst this study has observed a maximum mean crack length at 10.95 mm. It is expected, however, that on-bone cartilage experiences greater damage than off-bone cartilage; as previously hypothesised that the resulting energy may be released as cracks [13]. This is primarily as a result of the rationale of the presence of the underlying bone that provides constriction to the cartilage, increasing the induced stress [12–14]. This concept is further reinforced by the deep zone of articular cartilage restricted in its ability to deform laterally [41]. The ability of bone to dissipate more energy after an applied load than cartilage [42] is also in support of increased damage for on-bone cartilage.

Previous studies have identified the condition of “sclerotic subchondral bone” [43] at an increased BMD in volume associated with OA [44, 45]. Further, there is an established link of OA with a high BMD as reviewed [46] and extensively described elsewhere [7, 30, 47–56]. This study contributes to the outcome of these findings, such that it is not necessarily the change in BMD, alone that encourges cartilage damage, but that BMD interacts with other factors such as above normal-gait frequency. It is hypothesised that the remodelling process may account for further predisposition to damage [57].

The results indicate an increase in cartilage damage with substrate density, and therefore, the use of a softer substrate may redistribute stresses over a larger underlying area. Our study has modelled osteoarthritic to osteoporotic bone, using commercial grades of synthetic materials used to mimic bone as substrates, which has allowed assessment of cartilage failure across a range of substrate densities. This study is the first to illustrate the effects of the specific combination of BMD and an above-gait frequency, on cartilage damage, and therefore potentially to OA predisposition/progression. The resulting crack propagation through articular cartilage, may be worth investigating in future at 1 and 10 Hz.

### Limitations

It is worth highlighting the potential limitation of testing off-bone cartilage, due to the absence of the restrictive attachment provided by the underlying subchondral bone that is found in the natural environment in-vivo [14]. The removal of the subchondral bone creates an alteration in the load transfer properties of the cartilage-off-bone specimen, notably the absence of the calcified cartilage layer with a stiffness in-between that of articular cartilage and the subchondral bone [15]. Despite this, however, the substrates used in this study have acted as the underlying bone of a controlled density, to allow for evaluation of the effects of the density of the underlying substrate alone, on associated cartilage damage.

While the results for off-bone cartilage failure in this study are larger than for on-bone data in the literature [2], there are limitations in directly comparing the on- and off-bone cartilage results from this study and the previous study [2]. Although identical joint locations i.e. the bovine humeral head have been assessed, load ranges applied during testing have differed, as well as specimen geometries [2]. In addition, in this study bovine articular cartilage cores have been removed from the adjacent regions of cartilage, therefore, weakening the cartilage due to the disruption of its extracellular matrix. The previous study [2], tested a select area of cartilage on a large joint sample with an undisrupted matrix [58]. However, the removal of the articular cartilage in this way was kept consistent throughout the investigation, thus specifically concerning the unknown relationship between underlying substrate density and cartilage, off-bone, at a varied frequency.

This study has used freeze-thaw cycles when preparing samples for testing. Although this process may have limitations, a recent study has concluded “multiple freeze-thaw cycles cannot be explicitly or statistically linked to mechanical changes within the cartilage” [59]. Previous studies have utilised bovine humeral heads [22], bovine knee joints [23], as well as bovine femoral heads [25]; each study referring to the absence of a freeze-thaw effect on the mechanical properties of cartilage [26]. Additionally, the storage of tissue at − 40 °C is a previously established protocol utilised by several studies [20–25], and therefore was the approach taken for tissue storage in this study. Regardless, the results and conclusions obtained from this study on the effect of frequency and substrate on surface failure of cartilage are based upon a controlled testing protocol, and so findings are unlikely to be biased due to the freeze-thaw process used.

The use of an indentation test with articular cartilage is an established method previously developed to closely represent the physiological loading conditions of articular cartilage in vivo [60], as well as for damage inducing to the surface of articular cartilage [2]; having the advantage of being highly repeatable. Despite the hardness as well as the smaller diameter of the indenter in comparison to the cartilage specimen, a 0.5 mm radius bevelled edge was used to prevent artificial damage induced through stress concentrations at the edge. Further, the use of an indenter with a diameter smaller than the cartilage specimen, enables the deformation behaviour by the collagen matrix of the surrounding cartilage specimen [61] outside of the indentation area. Ultimately, this protocol has enabled a controlled evaluation of the effect of substrate density on cartilage failure.

## Conclusions

The effect of substrate density on the surface damage of articular cartilage-off-bone is multi-factorial. The significant increases in cartilage damage with increased substrate density, additionally requires the application of loading at 10 Hz in frequency. Peak surface damage was observed at 10 Hz, detected as multiple parallel lines of varying lengths; in contrast to single straight-line profiles produced at 1 and 50 Hz. Thus, the effect of bone mineral density on the onset of osteoarthritis, should also be considered with additional damage inducing factors, including an above-normal gait of frequency at loading.

## Abbreviations

BMD: Bone mineral density
OA: Osteoarthritis
